# Factors that Influence the Way Communities Respond to Proposals for Major Changes to Local Emergency Services: A Qualitative Study

**DOI:** 10.1371/journal.pone.0120766

**Published:** 2015-03-25

**Authors:** Helen Barratt, David A. Harrison, Naomi J. Fulop, Rosalind Raine

**Affiliations:** 1 Department of Applied Health Research, University College London, 1–19 Torrington Place, London WC1E 6BT, United Kingdom; 2 Intensive Care National Audit and Research Centre, 24 High Holborn, London WC1V 6AZ, United Kingdom; The University of York, UNITED KINGDOM

## Abstract

**Objective:**

According to policy commentators, decisions about how best to organise care involve trade-offs between factors relating to care quality, workforce, cost, and patient access. In England, proposed changes such as Emergency Department closures often face public opposition. This study examined the way communities respond to plans aimed at reorganising emergency services, including the trade-offs inherent in such decisions.

**Design:**

Cross-sectional study involving in-depth interviews. Participants selected their priorities for emergency care, including aspects they might be prepared to have ‘less’ of (e.g. rapid access) if it meant having ‘more’ of another (e.g. consultant-delivered care). A thematic analysis was carried out, combining inductive and deductive approaches, drawing on theories about risk perception.

**Setting:**

Two urban areas of England; one where changes to emergency services were under consideration (‘Greenville’), and one where they were not (‘Hilltown’).

**Participants:**

28 participants in total. Greenville interviewees included more common emergency service users - parents of young children (n=5) and older people (n=6) - plus patient representatives and individuals campaigning against service closures (n=9). Hilltown interviewees (n=8) received outpatient care for Chronic Obstructive Pulmonary Disease, an important cause of emergency admission.

**Results:**

Most participants, in both areas, were not willing to accommodate the trade-offs involved in consolidating emergency services, principally because of the belief that timely access is associated with better outcomes. Participants did not consider the proposed improvements as gains worth having; interviewees believed care quality would be adversely impact, partly because increased patient numbers would place staff under greater pressure and result in longer waiting times.

**Conclusions:**

Visible clinical leadership and detailed explanation of the case for change were insufficient to overcome opposition to the reconfiguration in Greenville, challenging the assumption that communities can be persuaded by evidence. Commissioners should make explicit credible plans to accommodate changes in patient flows, as well as clarifying the roles played by key staff groups.

## Introduction

Health care systems around the world face the challenge of meeting rising demand for care with diminishing financial resources.[1, 2] Attempts to tackle this dilemma commonly involve proposals to re-organise health care services and systems. The challenge for health care purchasers—known as commissioners in England—is to try to optimise the key issues of quality, safety, patient access, workforce and costs.[3] However, policy analysts have noted that compromises (or trade-offs) between these issues are unavoidable, for example between the quality and financial gains that may be achievable through the concentration of services on fewer sites, versus the social and clinical costs of reduced access for patients.[2] Moreover, different stakeholders are likely to prioritise different concerns. For example, managers may focus on value for money, whilst clinicians and professional bodies may prioritise quality and workforce issues,[4] and members of the public and patients may rank rapid access to care as their chief concern.[5, 6] For example, recent plans by commissioners to centralise paediatric cardiac surgery services in England in order to address quality concerns, prompted public opposition due to the distances that families would need to travel to access care and visit inpatients.[7]

Reorganisation plans often involve a range of services. However, it is proposals to alter Emergency Department (ED) services, that typically create the greatest public opposition.[4] In several areas of England, plans have been set out to reconfigure emergency care, partly in response to evidence that patients admitted to hospital out of hours have higher mortality rates. [8] Partly to address this, the College of Emergency Medicine recommends that hospitals should have a consultant present in the ED at least 16 hours per day, seven days per week. However, in England, current emergency consultant numbers are said to be ‘woefully inadequate,’ [9] requiring difficult and complex decisions to be made about how to reconfigure and structure local services appropriately.[10] One possible solution is to consolidate the resources of several EDs on fewer hospital sites. However, such proposals typically spark public concerns relating to the safety of centralised services and the potential risks that may be involved in having to travel further for care.[5, 6]

Spurgeon and colleagues argue that, in the context of the reconfiguration process, the differing emphases of various stakeholder groups may be irreconcilable.[11] This is not least because proponents and opponents of change appear to operate within different paradigms of understanding about risk. [6] Thus, the anthropologist, Mary Douglas observes that public perceptions about risk are often at odds with the ‘expert scientific’ view, because the public defines levels of risk in terms of the probability of an adverse event occurring (e.g. delayed access to care), the magnitude of potential repercussions of that event (e.g. death) and the value placed on that outcome. [12–14] However, she argues that the assessment depends largely on the value placed on the potential outcome: the public select a risk as worthy of their attention because they particularly value what is being threatened.[13] In fact, risk has loosened its links with probability to such an extent for the public that ‘risk’ now refers simply to the possibility of negative outcomes.[12, 15] She suggests that this is why public responses to potential risks often appear to be at odds with ‘expert’ scientific opinion.[14]

In contrast, other commentators argue that public opposition to reorganisation occurs because the case for change has been poorly articulated by commissioners [2, 3] or because of a ‘lack of public understanding of technical patient safety arguments.’ [4] Consequently, government documents increasingly emphasise the role of ‘evidence,’ clinical leadership, and better public consultation, apparently assuming that, if the public are presented with the ‘right evidence,’ they will be convinced of the need for change.[11]

The work of Spurgeon *et al* and Douglas suggests that the perspectives of all stakeholders need to be explored if we are to understand the factors that influence the way local communities may respond to proposals for major service change, such as a reorganisation of ED services. However, there is only a limited research literature examining the process of reorganising—or reconfiguring—hospital services, especially regarding the dynamics of local decision-making.[4] Two previous studies explored the views of a range of stakeholders.[4, 11] In both cases, however, only members of official patient involvement committees were interviewed, whose views may differ from other population groups.[16] We know little about the determinants of public opinion. This includes the extent to which local communities are prepared to accommodate the trade-offs involved in reconfiguration decisions,[6, 17] such as the potential benefits that may be gained by patients being treated by more specialised clinical teams, versus greater travel times because these teams operate from fewer centres.[2] In their study of the politics and process of hospital change, Farrington-Douglas and Brooks suggest that the views of patients and the public may differ in areas where reconfiguration is proposed and areas where it is not. They demonstrate that, whilst patient representatives and hospital campaigners often agreed with the general rationale for improving services, they opposed changes when services at their own local hospital were threatened.[4] However, we know little about the different ways in which the public may respond to proposals aimed at consolidating services on fewer sites.

This paper seeks to address this gap in the literature. We present the findings of a qualitative study exploring the factors that influence the way local communities may respond to proposals to reorganise local Emergency Department services, including the extent to which patients and the public may be willing to accommodate the trade-offs inherent in such decisions. We have focused on the reorganisation of emergency services, as this typically creates the greatest concern amongst local communities.[4] Risk is often central to public opposition. We therefore specifically sought to explore the ways in which the public understand risk in this context, and the role it plays in informing views about service change. To do this, we drew on theories of risk perception as an analytical focus, including Douglas’s work on the cultural role of risk.[12, 14, 15], Building on the work of Farrington-Douglas and Brooks, we also sought a broad range of different perspectives to examine whether views might be influenced by either personal characteristics (e.g. age, gender, health condition) or the existence of local proposals for service change. [4]

## Materials and Methods

In order to explore the factors that influence the way in which patients and the public respond to proposals aimed at reorganising ED services, semi-structured, one to one interviews were conducted—an approach which permits the in-depth exploration of each participant’s preferences, motivations and decisions.[18] To obtain a broad range of perspectives we sampled different population groups in different contexts. This included members of patient involvement committees, who have been the focus of previous research, but also members of the wider public, who may be likely to use ED services. We also compared views in two urban areas of England: one where changes to emergency services were under consultation, and another where they were not.

Economists make use of discrete choice experiments (DCEs) to understand preferences for particular goods or services, including the extent to which individuals are willing to trade one attribute for another. DCEs are a quantitative survey technique in which participants are asked to state their preferred option from a list of hypothetical scenarios. Although it provides information about the relative importance of the selected options, this approach provides limited scope for the researcher to explore why one option is considered more desirable than another.[19] Consequently the topic guide used for the interviews, drew on the principles of DCEs, but used qualitative methods to examine in detail the extent to which members of the public may be willing to accommodate the trade-offs said to be involved in reconfiguration decisions.[3] For example, in light of public concerns about the risks of travelling further for care,[4] we sought to explore the extent to which participants were willing to trade-off rapid access to the ED with other attributes of hospital emergency care, such as consultant-delivered services. During the remainder of the interview, we used this information as a prompt to explore how participants perceived and prioritised risk in the context of emergency care, including the role this played in their willingness to trade-off, using risk perception theory as an analytical focus.

### Study sites and interviewees

In the first study area, participants were all residents in an area referred to as ‘Greenville’. At the time, a public consultation was taking place locally about consolidating a range of hospital services on fewer sites, including emergency care. If the proposals went ahead, the ED at the local district general hospital would be replaced by an urgent care centre and residents would be required to travel to one of the two other hospitals in the area. The views of individuals from three groups were sought in Greenville: older people and parents of young children—both groups who may be likely to attend the ED;[20] and local ‘activists,’ including members of local patient involvement groups, and individuals campaigning against the closure of services.

In the second study area, the participants were NHS patients at a hospital we refer to as ‘Hilltown’, receiving outpatient care for Chronic Obstructive Pulmonary Disease, which is an important cause of emergency hospital admission in England.[21] Hilltown is a teaching hospital with an ED. At the time of the study, there were no public discussions taking place about reorganising services locally.

The study described in this manuscript formed part of a larger piece of research. This examined not only the ways in which patients and the public view the reorganisation of ED services, but also the factors that influenced a local community’s response to a consultation process designed to engage them in decision-making about reconfiguration proposals. A larger number of participants were therefore recruited in Greenville, the area where changes to hospital services were under consideration. For the research component described here, the sampling strategy aimed to include individuals with a range of experiences and backgrounds. We thus sought to recruit interviewees from the four subgroups described to explore variations within different population groups, in different contexts, where reconfiguration was proposed and where it was not.[22]

Participants' demographic characteristics are outlined in Table 1. Recruitment in Greenville was carried out via relevant community groups who were given details of the study and asked to help identify members who would potentially be willing to participate. This was typically done by email cascade, for example as part of a regular newsletter. A research nurse at Hilltown helped to identify eligible potential participants who would be attending the outpatient clinic during the study period, contacting them in advance by telephone to invite them to take part. All participants met the interviewer (HB) for the first time at their interview. Participants were informed that the interviewer was from a university, that the research was independent of local NHS structures, and that our primary motivation was to explore patient preferences and priorities for hospital emergency care.

**Table 1:** Participant demographic characteristics.

	Number of participants	Number of females	Number from Black and Minority Ethnic Groups	Age range
**Greenville Parents**	5	5	1	28–40
**Greenville Older Participants**	6	2	0	65–85
**Greenvile Activists**	9	4	2	60–76
**Hilltown Patients**	8	3	0	52–81
**Total**	28	14	3	28–81

Development of the interview topic guide drew on empirical literature regarding the reconfiguration process and conceptual literature about public perceptions of risk.[14, 15] An exercise exploring the extent to which the participant was prepared to accommodate trade-offs between different aspects of emergency care (‘the trade-off exercise’) formed an integral part of each interview. For this exercise, we used flash cards detailing different aspects of emergency care as the basis of the discussion (see Table 2). During the interviews, participants were invited to select their priorities for emergency care from the flash cards. This led on to a discussion about whether there were aspects of care listed on the cards that they might be prepared to trade-off, i.e. have ‘less’ of (e.g. rapid access to the ED) if it meant having ‘more’ of another (e.g. senior clinicians present in the ED). The interviewer then explored why the participant considered the chosen attributes of emergency care to be priorities, and the rationale behind their trade-off decisions. The development of the cards drew on literature about the reconfiguration process, as well as concerns raised by the public during a previous engagement exercise about reconfiguration proposals in another urban part of England.[17] The text of the guide was refined via pilot interviews, as well as discussions with NHS commissioners and patient representatives with experience of reconfiguration.

**Table 2:** Content of interview flash cards.

A local hospital to serve the local community
Good bus or tube links
Easy to park
Patients can choose which A&E to go to
Sick patients taken to A&E as fast as possible
Consultants on duty in A&E 24 hours a day
Patients’ care meets nationally agreed standards of quality
A&E convenient to get to for patients and their families

Face-to-face interviews took place between August 2012 and November 2012, usually at the interviewee’s home or, for the NHS patients, in the outpatient department at Hilltown Hospital. Interviews were conducted by HB (MBBS MSc) who was in a research training fellow position at the time. HB is female and has undergone training in qualitative interviewing and analysis, at Masters level. Interviews typically lasted between 45 minutes and one hour. Interviews were recorded and transcribed for analysis. Each participant was interviewed once. One interview was conducted with two participants; all others were individual. Interviewees were anonymised and details identifying the sites were removed. Participant study numbers are used here to set quotes in context.

### Analysis

A thematic analysis of the interview data was carried out, combining inductive and deductive approaches, drawing on theories about public perceptions of risk as an analytic focus, whilst at the same time allowing themes to emerge direct from the data. Pre-defined themes, derived from empirical and theoretical literature, included risk, safety and hospital reconfiguration proposals. These themes were expanded and refined inductively and new themes were also added to the initial framework.[18] A sample of the transcripts was read by the full research team (HB, DH, NF and RR) to identify and agree key themes, after which one researcher (HB) coded the interview transcripts. N-Vivo (version 10) was used to manage the analysis. Data were analysed within and between study groups.

### Ethics Statement

Ethical approval for the study was obtained from the East Midlands—Nottingham 2 Research Ethics Committee (REC reference number 12/EM/0258). All participants provided written informed consent before taking part.

## Results

In this section, we describe first the extent to which participants in Greenville and Hilltown were willing to accommodate the trade-offs inherent in decisions about reorganising emergency care. We then go on to describe the two main factors that influenced the way in which interviewees responded to proposals for major service change. Within this analysis, we illustrate the role of risk in informing responses to reconfiguration, as well as highlighting key differences and similarities between the different participant groups.

### Willingness to trade-off

Much of the discussion during the trade-off exercise focussed on potential compromises between timely access to emergency care, consultant-delivered care and care quality. Notably only two out of 28 participants—both Greenville residents—were prepared to consider a longer journey to hospital in an emergency, if it meant potential improvements to services. These two individuals were also the only Greenville interviewees who were broadly in favour of the proposed changes in that area. One—an older participant—had previously worked closely with the medical profession; the other was a member of a patient involvement group.

Most participants in both study areas were, however, not willing to accommodate the trade-offs assumed to be involved in decisions about whether to consolidate emergency care on fewer sites.[3] Some rejected the trade-off approach as overly simplistic (n = 5): they could not say whether they would be prepared to trade-off different aspects of care, because their response would depend on a number of variables, for example, the seriousness of the situation and the additional distance they would be required to travel.

Amongst the other interviewees, there were two distinct perspectives. The first perspective was held by a minority of participants (n = 2) who took issue with the concept of making trade-offs at all in the context of health care. Both these individuals were parent participants from Greenville. The views of one of them, Greenville Parent 4, illustrate this position well. When asked if she would be prepared to have a slightly longer journey to hospital, if there were to be a consultant in the ED when she got there, she responded:

*I suppose so… If it doesn't necessarily make me more sick or my life more threatened by travelling a bit longer, I would do it. But then again why should we have to? This is not something we should be made to choose…. Why should another hospital have better services in the first place? What they should be doing is upgrading the standards everywhere. It should be the same. This is the National Health Service. (Greenville Parent 4)*

Later in the interview, she added:

*I don't think trading off… We should never ever have to trade-off with national health services. It's okay to discuss it, but it's not something that should be considered. Why don't they just upgrade services all round? (Greenville Parent 4)*

The two participants who held this perspective were not prepared to contemplate trading-off any aspects of health care. It may be that they were unaware of purported workforce challenges currently faced by EDs, such as the reported challenge of recruiting sufficient consultants and middle grade doctors.[23] However, they were unwilling to accept the existence of any constraints on local services.

The second perspective on trade-off was held by the majority of participants, including participants in all four study groups, in both Greenville and Hilltown, and consequently is explored in detail in this paper. These interviewees were not willing to accept any trade-off of aspects of emergency care with access. Greenville Parent 2 illustrates this view. During the interview she expressed concern that there had not always been a consultant present, when she had taken her children to the Emergency Department in the past. However, when asked if she would be prepared to have a slightly longer journey, if she knew there would definitely be a consultant on duty, her response was clear:

*Er, no. Because in extreme circumstances that could be the matter of between life and death couldn’t it? In an extreme situation, you know, taking your child to hospital, minutes could be critical. So having a facility further away is… No, it’s not ideal at all…. If it was a critical time and minutes counted, then that would be redundant by the time you got there. [Getting there] is critical… I think having a facility nearby is essential, absolutely essential. (Greenville Parent 2)*

### Factor 1: Timely access to the Emergency Department

Greenville Parent 2 was not alone in her view about the importance of timely access. This represented the first of two main factors that influenced the way in which participants responded to proposals for major service change, particularly amongst those who were not willing to trade-off aspects of emergency care.

Interviewees in both geographical areas recognised that they would be ‘frightened’ (Hilltown Patient 8) or ‘worrying’ (Greenville Parent 1) in an emergency. Equally, whilst at home they would neither know ‘what the ramifications are,’ (Greenville Older Participant 1) nor know what to do for an unwell relative. On arrival at hospital, carers and family members are able to pass over responsibility or ‘the burden’ (Greenville Activist 1) to the ED staff.

However, for the majority of participants, regardless of their place of residence, there was also an implicit assumption that there is a direct association between timely access to emergency care and better outcomes. As Greenville Parent 2 explained in the quote above, many interviewees believed that, in an emergency, every minute counts. The importance of timely access lay not just in getting to the hospital, but in getting ‘to experts’ (Greenville Activist 1) and ‘getting seen quickly.’ (Hilltown Patient 7) Great emphasis was placed on the importance of the initial medical response in an emergency: time (or speed) ‘is of the essence’ (Hilltown Patient 6, Greenville Older Participant 1, Greenville Activist 2) because the problem requiring attention might get worse with time. Consequently, ‘the quicker I get to an Accident and Emergency with a serious problem, the better my chances.’ (Greenville Activist 9)

As we have noted, Douglas argues that risk has loosened its links with probability to such an extent for the public that it now refers simply to the possibility of negative outcomes.[12, 15] Indeed, participants in this study recognised that the likelihood or probability of them requiring a time critical intervention in an ED was remote: ‘the really big stuff happens thankfully less often and might not even happen at all.’ (Greenville Parent 3) However, because of the belief that timely access was associated with better outcomes, the magnitude of the potential outcome was considered more significant. In this way, risk played a key role in influencing the response of these participants to proposals for service change. Those who were unwilling to compromise on timely access to the ED, believed that travelling further for care could put their life in jeopardy, or the lives of their loved ones. As Douglas suggests, this potential risk was identified as particularly important, because of what was under threat.[13] The fact that access *could* be delayed, with potentially devastating consequences, was more important than whether or not it was likely to occur. Consequently, having to travel further to access care constituted what Douglas describes as ‘unacceptable danger.’[14]

### Factor 2: Impact of reorganisation on Emergency Department care quality

Whilst Greenville Parent 2 expressed concern that there had not been a consultant present in the ED during her previous visits, others were not prepared to compromise on access to gain greater levels of consultant-delivered care. This was because they did not consider the anticipated improvements in technical aspects of emergency care, such as this, to be gains worth having. In fact, many in both Greenville and Hilltown believed that plans to consolidate care on fewer sites would negatively impact care quality. This represented the second main factor that influenced the way in which the majority of participants responded to proposals for major service change, and hence their willingness to trade-off aspects of emergency care.

For clinicians and commissioners, care is typically evaluated on grounds of its technical quality, for example patient outcomes and practice consistent with current professional knowledge.[24] However, most participants in this study assessed quality in terms of their interactions with ED staff—what Donabedian refers to as the interpersonal quality of care.[25] This included both the timeliness of the care and the attentiveness of staff. High quality emergency care constituted two things: ‘not having to sit around for hours’ (Greenville Older Participant 1) and ‘having plenty of staff around.’ (Greenville Parent 5)

For many, there was a degree of resignation about waiting in the ED. Others, however, expressed concern about the potential ramifications of lengthy waiting times. This was partly because patients attending an ED are likely to be in discomfort or pain. Linking back to the belief that timely access is associated with better outcomes, there was also a fear that a patient’s condition might deteriorate unnoticed, if they have to wait unduly. This concern therefore also links to the perceived risks involved in service reconfiguration, specifically the importance placed on timely access to medical care. Some also felt that long waiting times indicate that patients are not being attended to appropriately, or that the ED staff are in some way uncaring. A previous lengthy wait in an ED had left one participant wondering, ‘are they looking after us properly?’ (Hilltown Patient 1)

Reflecting these anxieties, future waiting times were a source of concern for many participants in Greenville, where the closure of the local ED was being discussed. The public consultation document provided by the commissioners focussed on the clinical rationale for change. It provided very little information about the changes that would be made to the remaining services, for example, measures to expand facilities to accommodate increased patient flows. Perhaps consequently, interviewees perceived that, if the local ED were closed, ‘double’ the number of patients would attend the nearest alternative hospital, with a knock-on effect on waiting times. (Greenville Older Participant 4) Participants also believed that increased patient flows would put even greater pressure on ED staff. Some shared the view that hospitals are already ‘swamped’ with patients. (Hilltown Patient 6) Consequently, an increase in patient numbers would not only result in longer waits, but would also mean staff having less time to give to individual patients, negatively impacting the quality of care.

The interview data suggest two possible reasons why the majority of participants did not consider potential improvements in technical aspects of emergency care to be ‘gains’ worth having, at the expense of timely access. First, the Greenville proposals were partly aimed at addressing variations in care locally. However, it was clear that participants in both study areas perceived there to be little difference in terms of the care currently provided by different Emergency Departments. This was mainly because, for most, care standards largely related to the knowledge and skills of the staff, rather than the technologies available to them. There was an expectation that all hospitals would be broadly similar:

*As far as I’m concerned, all hospitals should be the same… The doctors have all gone through their courses and done their five or seven years, so whether you’re in Leeds or in London, it should be the same. (Hilltown Patient 6)*

Second, for the commissioners, a perceived need to increase levels of consultant-delivered care in EDs represented another driver behind the Greenville proposals. However, aside from a small number of interviewees who were actively campaigning against the plans, none of the participants in Greenville linked this issue with the case for change there. When asked, most interviewees in both study areas were not able to describe the role of consultants in the ED. Whilst some felt there would be advantages to having experienced staff around, others felt that this was not necessary in every situation:

*I am fairly confident that most doctors would be able to handle what was going to be presented to them. (Greenville Parent 3)*

Equally, there were participants who were clear in the view that consultants would not be present in the ED. They perceived consultants to be specialists in caring for particular body systems or patient groups, for example, children, and distinct from ED doctors who are general ‘resuscitators.’ (H5) A patient therefore would not see a consultant in ED, but would be referred on to a specialist once the ED staff had stabilised their condition.

In contrast, the activists did acknowledge the emphasis the commissioners placed on consultant-delivered care, but disputed its importance:

*I don’t accept the argument that they use that you have to have a consultant… My understanding is [that] you just need to have good experienced A&E doctors and health care professionals to attend to you. (Greenville Activist 9)*

The activists argued strongly that Greenville needed a local hospital, partly because many believed that the state had a duty to provide communities with a comprehensive local health service. To have acknowledged that the care currently provided by that hospital was less than adequate, would inevitably have undermined their argument that no change was needed and services should remain the same. Those actively campaigning against reconfiguration proposals are often most heard in debates.[16] However, in Greenville, the views of the activists were not entirely representative of the community at large: the wider public were, in contrast, generally much less familiar with the details of the case for change.

## Discussion

In this study, we explored the factors that influence the way communities may respond to proposals to reorganise local Emergency Department services, including the extent to which patients and the public may be willing to accommodate the trade-offs inherent in such decisions. Two major factors were identified: 1) the importance placed on timely access to the ED, because of the perceived risks of delayed treatment and 2) the anticipated impact of service changes on the quality of care.

Most participants were not willing to accommodate the trade-offs involved in decisions to consolidate emergency services on fewer sites. This was principally because of the widespread belief, held across the study population, that timely access to the ED is associated with better outcomes. Using Douglas’s work on the cultural role of risk as a theoretical focus helped explain the significance of this belief, demonstrating the importance of risk in this context. Participants’ perceptions of the risks involved in service change appeared to be at odds with expert scientific opinion, because they interpreted risk in a different way.[14] Interviewees recognised that there was a low probability of them suffering a medical event that required a time critical intervention. However, because most believed that timely access is associated with better outcomes, the magnitude of the potential outcome (death), should delays be incurred, was much more important.[14] Consequently participants in both study areas were not prepared to contemplate a longer journey to hospital in an emergency. Alongside concerns about the risks of consolidating services on fewer sites, participants did not regard the anticipated improvements in technical aspects of emergency care, such as consultant-delivered services, to be gains worth having. In fact, interviewees believed such plans would negatively impact care quality, because increased patient numbers at the remaining EDs would result in greater pressure on staff and longer waiting times, thereby delaying timely access to care. In this way, perceptions about risk also played a role in informing this second area of concern.

Our goal was to obtain a broad range of perspectives, rather than providing a representative study of public opinion. Nevertheless, it is noteworthy that participants in all four study groups, were unwilling to accept a longer journey to hospital in an emergency. Underpinning this was the widely held belief that delayed access, which could potentially result in death, constituted an unacceptable danger. Participants’ interpretation of the potential risks involved in reconfiguration did not appear to be significantly affected by whether or not local emergency services were under threat: views about timely access were similar in both areas. However, this warrants further study. Views about the anticipated improvements in technical aspects of emergency care were also similar across the study population, with many participants in both areas arguing that service consolidation would negatively impact care quality. As we highlighted, the key point of difference between the activists and other study participants was with regard to the detail of the clinical rationale for reorganising care. For most participants, care standards largely related to the knowledge and skills of the staff, and there was little sense that hospitals might differ in this regard. In contrast, the activists were much more engaged with the content of the case for change, including the purported need for consultant-delivered care in the ED. However, they disputed the rationale behind the proposals. This was perhaps because participants in this group argued that Greenville needed a local hospital, not least because of the belief held by many of them that the state should provide communities with a comprehensive local health service.

This study provides for the first time a detailed exploration of the factors that may influence the way in which local communities respond to proposals aimed at consolidating emergency care on fewer hospital sites. By examining the response through the lens of risk perception theory, we are able to offer a range of novel insights about how the public assess the potential implications of such plans. Whilst the literature indicated that timely access was likely to be a concern,[4, 6] little has been written about how the public evaluate the quality implications of major service changes such as this. Nevertheless, our findings are based on a relatively small number of interviews, conducted in only two geographies. Both of these were urban areas, and in only one was service change being considered. Our results may therefore be less relevant in other settings, for example rural areas, where access may be even more of an issue. It is possible that other contextual factors may have influenced participants’ responses to the notion of consolidating emergency services on fewer sites. For example, Greenville is in the most deprived 20% of local authorities in the country, whilst Hilltown Hospital’s catchment area includes a diverse urban population with significant pockets of deprivation. Demographic factors such as deprivation may impact patients’ health-seeking behaviours in an emergency, as well as their willingness to travel for care.[26] In addition, it is also feasible that the nature, reputation and perceived quality of existing emergency services may influence a community’s response to proposed changes, although this was not explicitly explored in this study. It is worth noting, however, that the concerns raised about service reconfiguration in Greenville echo those raised previously by residents in a range of other parts of the country, faced with similar changes.[2]

No previous studies of major service reorganisation have examined the views of the wider public. In addition, it was not known whether priorities varied within different population groups, or in different settings. This study sought to address these gaps. Drawing on risk perception theory has enabled us to demonstrate why local communities and commissioners appear to operate in different paradigms of understanding about risk, when a reconfiguration is being discussed.[6] Farrington-Douglas and Brooks observed that, whilst patient representatives often agreed with the need to change services *per se*, they opposed changes to their own local hospital.[4] However, most participants in our study viewed the consolidation of services in a negative light, including those interviewed in Hilltown. Concerns about having to travel further for care in an emergency, as well as the emphasis placed on the interpersonal quality of care, were shared across the groups. Our findings may have been influenced by asymmetry in sample sizes between Greenville and Hilltown, as well as the heterogeneity in the participant groups interviewed in each area. However, the fact that such similar views were held in Greenville and Hilltown suggests that such perceptions may be widely held by the public, and were not just a response to the proposals aimed at downgrading local services in Greenville.

In Greenville, visible clinical leadership and detailed explanation of the case for change were insufficient to overcome the opposition expressed by many participants in this study. Although based on a relatively small number of interviews, our findings suggest that this approach may not be sufficient to persuade communities to accommodate service reorganisations such as this which may compromise timely access to care in an emergency.[11] Whilst it seems unlikely that concerns about timely access could be completely allayed, the study has identified a number of ways in which communication could be improved. For example, commissioners should make explicit credible plans to accommodate greater patient numbers at the remaining EDs. We have also demonstrated a lack of clarity amongst patients and the public about technical aspects of care, such as the role of consultants in the ED. For example, if commissioners are seeking to communicate workforce reasons for change, they should first make clear the role of ED consultants and the way in which they are distinct from junior staff.

The risk of travelling further for care in an emergency is a significant concern for the public, but the evidence about this remains equivocal.[27] However, a quantitative study to explore this further would require the involvement of large numbers of patients from multiple centres, making it challenging and probably unfeasible. There is, however, scope for further research to better understand how patients and the public assess the quality and safety of the services they receive, and hence their priorities when changes are being considered. For example, our findings suggest that many participants did not consider consultant-delivered care in the ED important, perhaps because of a lack of clarity about the role of senior doctors. Additional qualitative research involving either focus groups or individual interviews, exploring patient priorities for services beyond the ED, could also provide useful information for those seeking to implement service reorganisations in the future.
